# Hemophagocytic Lymphohistiocytosis Associated With Epstein-Barr Virus Infection in an Immunocompetent Adult

**DOI:** 10.7759/cureus.75279

**Published:** 2024-12-07

**Authors:** Rafael Pinheiro Ramos, Joana Névoa, João Campos Cunha, Gonçalo Sarmento

**Affiliations:** 1 Department of Internal Medicine, Unidade Local de Saúde Entre Douro e Vouga, Santa Maria da Feira, PRT

**Keywords:** epstein-barr virus, hemophagocytic lymphohistiocytosis, hepatosplenomegaly, hyperferritinemia, infectious mononucleosis

## Abstract

Hemophagocytic lymphohistiocytosis (HLH) is a rare but potentially fatal entity characterized by an unregulated activation of the immune system. In the adult population, it is most commonly secondary to infectious, autoimmune, or neoplastic diseases. We present a case of a 23-year-old female diagnosed with infectious mononucleosis and hospitalized due to a persistent three-week fever and malaise with a new onset of jaundice and findings compatible with acute hepatitis and hepatosplenomegaly. Due to a lack of response to standard supportive care, maintained fever, persistent analytical worsening (hyperferritinemia, bicytopenia, cytocholestasis, and hypertriglyceridemia), and hemophagocytocic phenomena on myelogram, the diagnosis of HLH secondary to Epstein-Barr virus infection was attained. Treatment with systemic corticosteroids was initiated with a continuously favorable evolution.

This case aimed to describe and highlight the complexity of the diagnostic approach and the urgency of directed therapeutic attitudes in the management of HLH.

## Introduction

Hemophagocytic lymphohistiocytosis (HLH) is a rare but potentially fatal clinical syndrome consequent to an unregulated activation of the immune system leading to a cytokine storm, associated with extreme systemic inflammation and multi-organ dysfunction. It is thought to be consistently underdiagnosed, not only due to nonspecific symptoms and analytical findings but also due to an important overlap with much more common inflammatory syndromes such as sepsis [1,2].

HLH is classically divided into familial/primary and non-familial/secondary HLH. Primary HLH (pHLH) usually presents in younger patients (infants and young children) with identifiable genetic predisposition. On the other side, secondary HLH (sHLH) mostly occurs in adults with no clearly traceable predisposing genetic trait and is typically associated with infectious, autoimmune, or malignant processes. Nonetheless, it has been proven in recent years that the gap between these two entities is not as clear as previously thought, considering the likely genetic contribution and its interaction with external factors in all forms of HLH [1-4]. 

In this study, we present the diagnostic and therapeutic approach as well as the evolution of a young adult with HLH secondary to Epstein-Barr virus (EBV) infection. The aim of this study was to contribute to the characterization of this rare and complex syndrome and raise awareness for its required methodical approach.

## Case presentation

A 23-year-old female, physical therapist, with no relevant past medical history and medicated with an oral contraceptive, was admitted to the emergency department with a three-week history of persistent headache (frontotemporal bilaterally, without nausea/vomits, photo/phonophobia, or visual disturbances), myalgia, and fever (maximum temperature 38.5ºC with partial response to antipyretics). Since the onset of the described complaints, the patient has already been four times to the emergency department.

It was initially assumed (one week after the symptomatic onset) that the culprit was a lower respiratory tract infection, and the patient was discharged with a prescription for a three-day azithromycin course. Due to the aggravation of symptoms with the persistence of fever, the patient returned the following day. A cranial computed tomography (CT) was performed with no abnormalities, but the laboratory findings revealed leukopenia and thrombocytopenia, a slight increase in aspartate transaminase, alanine transaminase, lactate dehydrogenase, and C-reactive protein. These were interpreted in the context of a probable systemic non-specific viral infection, and she was discharged again under symptomatic treatment. The patient returned the following week due to worsening symptoms. A normal neurological examination and cerebrospinal fluid analysis ruled out central nervous system infection. However, there was a continuous increase in cytocholestasis and inflammatory parameters, as well as hyperbilirubinemia, not previously present. An abdominal ultrasound ruled out hepatic obstruction. The serological study displayed IgM positivity both for EBV and cytomegalovirus (CMV). Therefore, the diagnosis of infectious mononucleosis was established, and she was once more discharged under symptomatic treatment.

**Figure 1:**
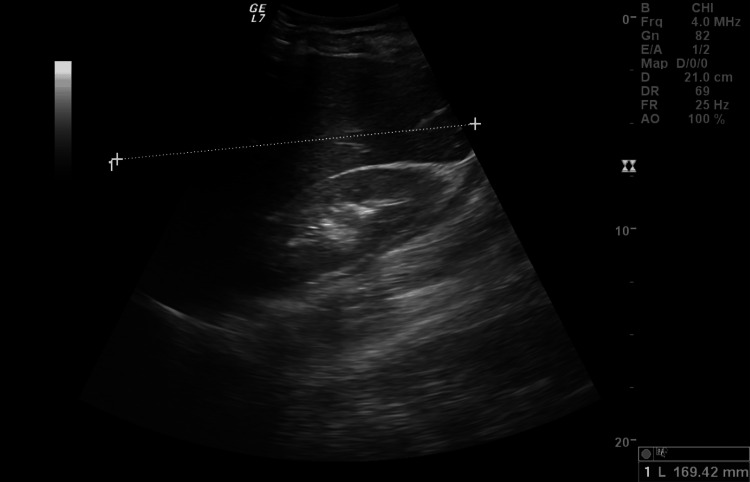
Hepatomegaly on abdominal ultrasound. Craniocaudal length: 169.42 mm

Finally, the patient returned to the emergency department three days later due to continuous symptomatic worsening, now accompanied by jaundice and generalized pruritus. We highlight the high value of ferritin (32073.86 ng/mL) and the ongoing increase in cytocholestasis enzymes and bilirubin (total bilirubin 8.16 mg/dL, direct bilirubin 6.09 mg/dL, and indirect bilirubin 2.07 mg/dL) (Table 1). A new abdominal ultrasound was performed with findings compatible with acute hepatitis and hepatosplenomegaly (Figure 1).

**Table 1:** Analytical parameters at admission and discharge.

Analytical parameters	Reference range/unit	Value at admission	Value at discharge
Hemoglobin (g/dL)	12.0-16.0	13.2	11.5
Leucocytes (x10^9^/L )	4.0-11.0	10.4	6.5
Platelets (x10^9^/L)	150-400	99	210
Ferritin (ng/mL)	4.63-204.00	32073.86	6859.29
Triglycerides (mg/dL)	<150	331	272
Aspartate transaminase (U/L)	5-34	354	170
Alanine transaminase (U/L)	0-55	487	412
Gamma-glutamyl transferase (U/L)	<38	322	307
Alkaline phosphatase (U/L)	40-150	562	437
Total bilirrubin (mg/dL)	0.20-1.20	8.16	2.67
Lactate dehydrogenase (U/L)	125-220	1572	-
C-reactive protein (mg/L)	<5.0	13.6	<1.0

Considering the persistence and difficult management of symptoms, the patient was admitted to the internal medicine ward for continuity of treatment. Initially, signs of symptomatic improvement were observed, nonetheless inconsistent with persistent fever and worsening cytocholestasis parameters. Concomitantly, she developed a pruritic maculopapular exanthema on the torso, back, and abdomen.

**Figure 2:**
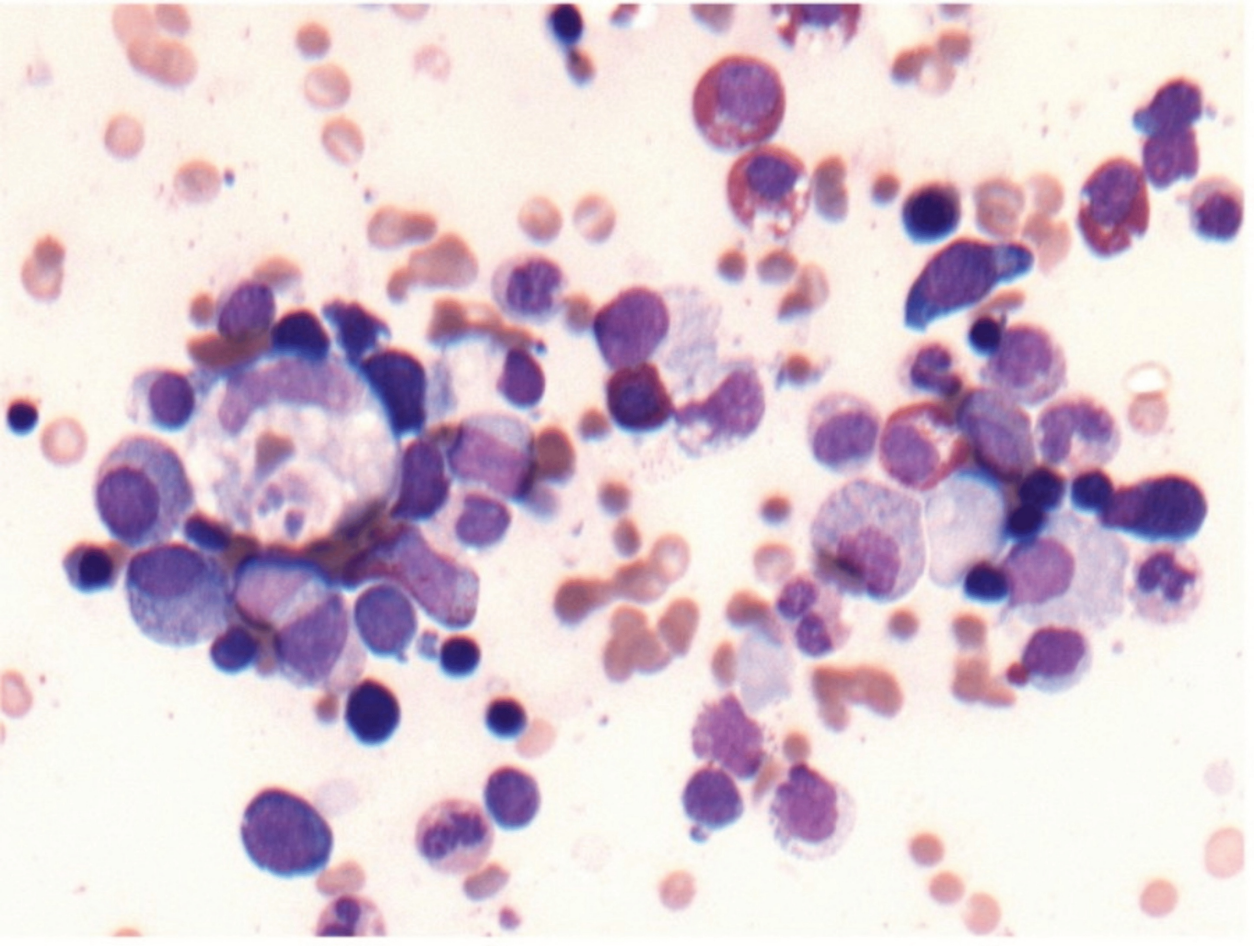
Hemophagocytocic phenomenon on bone marrow aspirate (400x magnification).

Due to the atypical course of disease, with persistent fever, hyperferritinemia, bicytopenia (anemia and thrombocytopenia), and hypertriglyceridemia as well as hepatosplenomegaly, suspicion of hemophagocytic lymphohistiocytosis arose. In articulation with hematology, bone marrow aspiration and biopsy were performed with evidence of hemophagocytosis, fulfilling five out of eight HLH-2004 criteria and a greater than 99% probability of HLH according to HScore (253 points) (Figure 2).

Considering the compatible clinical and analytical presentation, supported by concordant results from HLH-2004 and HScore and a later positive quantification of EBV viral load (2704 copies/mL of plasma log 3.43), the diagnosis of sHLH in the context of EBV infection was established.

**Figure 3:**
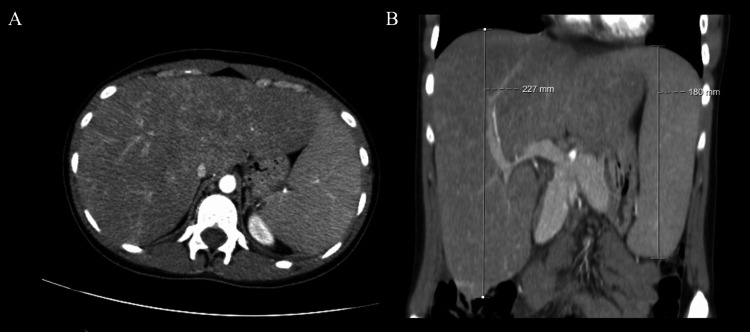
Hepatosplenomegaly on abdominal CT scan. A: axial plane; B: coronal plane

An urgent thoracic/abdominal/pelvic CT scan was performed with no unexpected findings besides the already described hepatosplenomegaly (Figures 3A, 3B). The patient was then started on dexamethasone (10 mg/m^2^) leading to sustained clinical and analytical improvement, with a decrease in inflammatory and cytocholestasis parameters, bilirubin, and ferritin levels. The extended immunological and serological study did not point towards another etiology besides EBV infection, as discussed below (Table 2).

**Table 2:** Immunological and serological tests during hospitalization. HIV: human immunodeficiency virus; EBNA: Epstein-Barr nuclear antigen; VCA: viral capsid antigen; DNA: deoxyribonucleic acid; IGs: immunoglobulins; ANAs: antinuclear antibodies; ANCAs: antineutrophil cytoplasmic antibodies

Analysis	Value/interpretation
Hepatits B serology	Immune
Hepatitis C serology	Negative
HIV serology	Negative
Epstein-Barr EBNA IgG	Negative
Epstein-Barr VCA IgG	Negative
Epstein-Barr VCA IgM	Positive
Epstein Barr DNA (copies/mL)	2704
Anti-CMV IgG	Negative
Anti-CMV IgM	Positive
CMV DNA	Negative
Immunoglobulin A	Normal
Immunoglobulin G	Normal
Immunoglobulin M (mg/dL)	587 (33-293)
Protein electrophoresis	IGs polyclonal increase
C3 complement	Normal
C4 complement	Normal
ANAs	Negative
ANCAs	Negative

**Figure 4:**
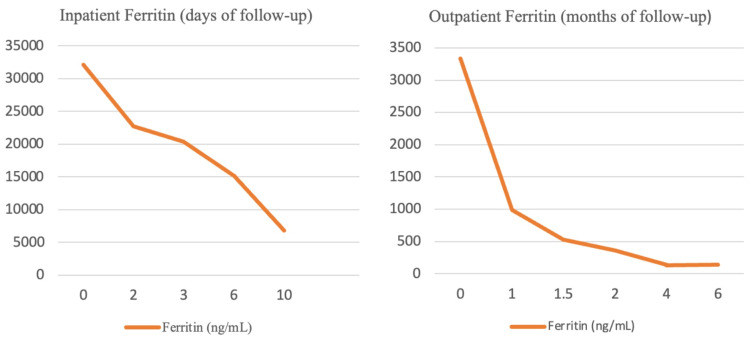
Evolution of ferritin value.

After nine days of hospitalization, the patient was discharged with an indication to complete a seven-week treatment course with dexamethasone, in a weaning scheme. Additionally, on account of iatrogenic immunosuppression, *Pneumocystis jirovecii* infection and glucocorticoid-induced osteoporosis prophylaxis were initiated. Follow-up was maintained through daily monitoring via an innovative online monitoring system of the National Health Service and regular evaluation in an outpatient setting. Since discharge and in the first year after hospitalization, we report a sustained favorable evolution with no signs of relapse and complete recovery both in clinical and analytical parameters, best exemplified by the sustained decrease in ferritin value (Figure 4).

## Discussion

EBV is a common human herpesvirus, considered to have infected at least 90% of the world population, mainly through salivary transmission and most frequently during childhood [5]. Primary EBV infection in young children is typically mild or asymptomatic, while in teenagers and adults, it may be responsible for infectious mononucleosis. After the acute phase of infection, the virus usually remains latent in B lymphocyte populations (memory cells) and, depending on host factors (associated with the disturbance of the immune system), may later reactivate and potentially contribute to the occurrence of autoimmune and neoplastic diseases [6]. EBV infection can be the trigger of HLH in patients with an identifiable inherited predisposition, the abovementioned primary HLH, as well as secondary HLH, in which it is seen as the most common infectious trigger [6-8]. In this context, sHLH may be non-neoplastic in its etiology, uniquely associated with the viral infection (EBV-HLH), or it could be related to an underlying lymphoproliferative process (mostly T/NK-cell chronic active EBV infection or T/NK-cell lymphoma) associated with faster disease progression, higher mortality rate and requiring a different therapeutical approach. Despite that, the clinical presentation of both EBV-HLH and EBV-associated lymphoproliferative disorders is overly similar, which highlights the importance of an adequate diagnostic strategy [7,9].

Regarding our patient, even though she could have inherited genetical defects that predispose her to HLH, it is unlikely that this would be the main factor for the onset of this syndrome given her age (pHLH usually occurs in younger patients) and concomitant acute EBV infection (not the main infectious trigger in pHLH, but clearly the main one in sHLH). Additionally, the excellent response to treatment goes in favor of a secondary etiology since allogeneic hematopoietic stem cell transplantation is required for permanent cure in pHLH [1,10,11]. Despite the relative certainty in the established diagnosis, given the possible association with an underlying malignant or autoimmune process (even in the presence of EBV infection), the etiologic study was broadened to exclude concomitant infections, autoimmune or neoplastic diseases [7,12]. As for the positive anti-CMV IgM, it was determined to be a false positive result, as the CMV viral load was undetectable, most likely due to the presence of interfering substances such as heterophile antibodies, a phenomenon documented in patients with acute EBV infection and both EBV and CMV IgM positivity [13].

The proposed treatment with dexamethasone was based on the available literature, bearing in mind the assumption of EBV-HLH in a clinically stable patient. Combination therapy with rituximab could have been considered but ultimately monotherapy was maintained, due to the excellent response to dexamethasone and the fact that the quantification of EBV viral load was only attained two weeks into treatment, at a stage where the patient was quickly improving [7,11,14].

Our patient had a favorable evolution with complete resolution of symptoms, normalization of analytical parameters, and progressive decrease in EBV viral load to undetectable values after seven months. Clinical vigilance may loosen but will always be required, with a lower threshold for secondary etiologic investigation in case of similar or suspicious non-specific clinical presentations [6,7].

## Conclusions

This study illustrates how a high level of suspicion is required in the diagnostic approach to HLH, as well as the impact of delays in initiating therapy. HLH is a deadly syndrome with multiple associated disorders, requiring a broad etiologic study. The proposed diagnostic criteria (HLH-2004, originally designed for pediatric patients) are controversial in adults, having led to the creation of the HScore, which may better support the diagnostic approach. Nonetheless, clinical judgment remains the key diagnostic element, considering that timely initiation of treatment is essential for a favorable outcome.
